# Supplementary material for: Sex Differences in MASLD After Age 50: Presentation, Diagnosis, and Clinical Implications
Source: Biomedicines. 2025 Sep 18;13(9):2292. doi: 10.3390/biomedicines13092292 (PMC12467267; doi:10.3390/biomedicines13092292)
Supplement: Supplementary file 1 [file biomedicines-13-02292-s001.zip › Table S1.pdf]

Table S1. Contingency tables (FLI vs. CAP ≥ 248) and chi-square test results, stratified by sex

Sex	**CAP ≥ 248**	**FLI ≤ 60**	**FLI ≥ 60**	**Overall**
Men	0	7	3	10
	1	3	43	46
Women	0	8	20	28
	1	9	83	92

For men, the Pearson chi-square test yielded $\chi^2 = 22.565$, df = 1, $p < 0.001$.

For women, the Pearson chi-square test yielded $\chi^2 = 6.232$, df = 1, $p = 0.013$.
