# Supplementary material for: Sex Differences in MASLD After Age 50: Presentation, Diagnosis, and Clinical Implications
Source: Biomedicines. 2025 Sep 18;13(9):2292. doi: 10.3390/biomedicines13092292 (PMC12467267; doi:10.3390/biomedicines13092292)
Supplement: Supplementary file 1 [file biomedicines-13-02292-s001.zip › Table S2.pdf]

Table S2. Multivariate analysis of variance (MANOVA) on the dependent variables (CAP and LSM).

Effect	Test Type	Dependent Variable	F	df	p-value	Partial η²
**Sex**	Multivariate		3.852	2	**0.023**	0.036
	Univariate	CAP	6.580	1	**0.011**	0.031
	Univariate	LSM	2.036	1	0.155	0.010
**Diabetes**	Multivariate		4.650	2	**0.011**	0.043
	Univariate	CAP	1.251	1	0.265	0.006
	Univariate	LSM	8.844	1	**0.003**	0.041

F-values (F), degrees of freedom (df), significance levels (p-value), and partial eta-squared (η^2) are reported for each effect. The effects of the other confounders (age, smoking, hypertension, dyslipidemia) were not statistically significant.

The multivariate analysis of variance (MANOVA) performed to examine the significant effect of sex on two dependent variables (CAP and LSM), adjusted for potential confounders revealed a significant multivariate effect of sex on the combined dependent variables *(F(2, df)* = 3.852, *p* = .023, partial η^2 = .036), as well as a significant effect of diabetes (*F(2, df)* = 4.650, *p* = .011, partial η^2 = .043). Univariate tests indicated that sex had a significant effect on CAP (*F*(1, df) = 6.580, *p* = . 011, partial η^2 = .031), whereas diabetes significantly affected LSM (*F*(1, df) = 8.844, *p* = .003, partial η ² = .041). Other effects (age, smoking, hypertension, dyslipidemia) were not statistically significant.
