# Supplementary material for: Sex Differences in MASLD After Age 50: Presentation, Diagnosis, and Clinical Implications
Source: Biomedicines. 2025 Sep 18;13(9):2292. doi: 10.3390/biomedicines13092292 (PMC12467267; doi:10.3390/biomedicines13092292)
Supplement: Supplementary file 1 [file biomedicines-13-02292-s001.zip › Table S3.pdf]

Table S3. Logistic Regression Model for the Association Between FLI and CAP ≥ 248 adjusted for confounders

		B	Sig.	Exp(B)	95% CI per EXP(B)	
					Lower	Upper
**Women**	*FLI*	*.033*	*.007*	1.034	1.011	1.057
	Diabetes	.455	.386	1.576	.609	4.083
	Dyslipidemia	-.287	.580	.750	.293	1.924
	SBP	.017	.292	1.018	.988	1.048
	DBP	-.015	.490	.985	.948	1.024
	Smoking	.102	.748	1.107	.624	1.963
	Age	-.051	.162	.950	.889	1.015
**Men**	*FLI*	*.109*	*.001*	1.115	1.053	1.181
	Diabetes	*.843*	.520	2.324	.216	22.01
	Dyslipidemia	*-1.820*	*.179*	.162	.014	1.887
	SBP	*-.064*	*.101*	.938	.875	1.007
	DBP	*-.049*	*.309*	.953	.874	1.039
	Smoking	*-.763*	*.208*	.466	.155	1.398
	Age	*.226*	*.051*	1.254	1.016	1.547

SBP =Systolic Blood Pressure; DBP= Diastolic Blood Pressure

For women: -2 Log Likelihood = 116.754, Cox & Snell R^2 = 0.083, Nagelkerke R^2 = 0.125. For men: -2 Log Likelihood = 25.720, Cox & Snell R^2 = 0.381, Nagelkerke R^2 = 0.625.

AUC Women= 0.680 (95% CI: 0.563-0.796, p = 0.005)

AUC Men= 0.920 (95% CI: 0.814-1.000), p < 0.001)

Inclusion of confounders in the logistic regression model for FLI vs. CAP showed that FLI retained a significant association even after adjustment for major confounders (diabetes, dyslipidemia, hypertension, smoking, age), with estimated coefficients significantly higher in men than in women. The confounders included in the model were not significant. The addition of these covariates also showed an improvement in the fit parameters (-2 log likelihood and R^2) and overall predictive accuracy of the model AUC in men (from 0.863 to 0.920, p=0.005), confirming a greater predictive capacity in this subgroup independent of the other factors considered.
