# Supplementary figures and images for: Sex Differences in MASLD After Age 50: Presentation, Diagnosis, and Clinical Implications
Source: Biomedicines. 2025 Sep 18;13(9):2292. doi: 10.3390/biomedicines13092292 (PMC12467267; doi:10.3390/biomedicines13092292)

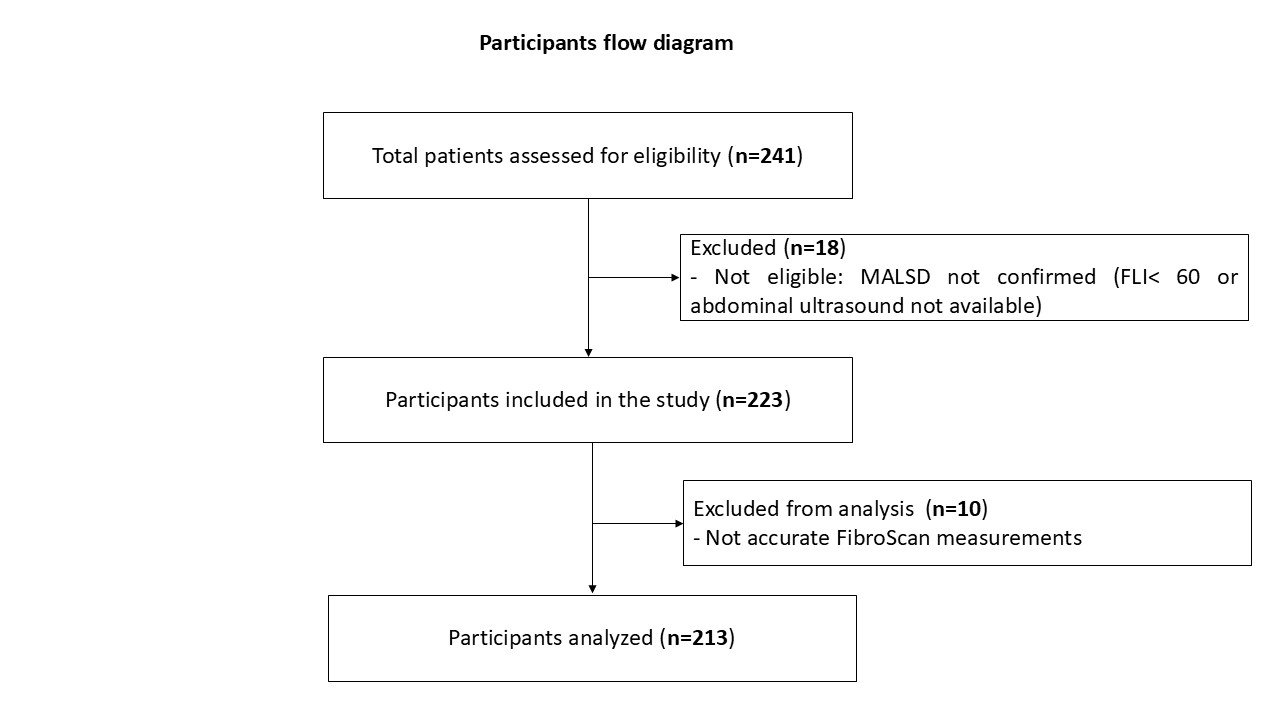

Supplement: Supplementary file 1 [file biomedicines-13-02292-s001.zip › Participants flow-diagram Fig S1.jpg]
